# hI-con1, a factor VII-IgGFc chimeric protein targeting tissue factor for immunotherapy of uterine serous papillary carcinoma

**DOI:** 10.1038/sj.bjc.6605760

**Published:** 2010-08-10

**Authors:** E Cocco, Z Hu, C E Richter, S Bellone, F Casagrande, M Bellone, P Todeschini, G Krikun, D-A Silasi, M Azodi, P E Schwartz, T J Rutherford, N Buza, S Pecorelli, C J Lockwood, A D Santin

**Affiliations:** 1 Department of Obstetrics, Gynecology and Reproductive Sciences, Yale University School of Medicine, Room 305 LSOG, 333 Cedar Street, PO Box 208063, New Haven, CT 06520-8063, USA; 2 Department of Pathology, Yale University School of Medicine, New Haven, CT, USA; 3 Department of Obstetrics and Gynecology, Division of Gynecologic Oncology, University of Brescia, Brescia, Italy

**Keywords:** uterine serous papillary cancer, hI-con1, immunotherapy, endometrial carcinoma

## Abstract

**Background:**

Uterine serous papillary adenocarcinoma (USPC) is a highly aggressive variant of endometrial cancer. Human immuno-conjugate molecule (hI-con1) is an antibody-like molecule targeted against tissue factor (TF), composed of two human Factor VII (fVII) as the targeting domain, fused to human immunoglobulin (Ig) G1 Fc as an effector domain. We evaluated hI-con1 potential activity against primary chemotherapy-resistant USPC cell lines expressing different levels of TF.

**Methods:**

A total of 16 formalin-fixed, paraffin-embedded USPC samples were evaluated by immunohistochemistry (IHC) for TF expression. Six primary USPC cell lines, half of which overexpress the epidermal growth factor type II (HER2/neu) receptor at 3+ levels, were assessed by flow cytometry and real-time PCR for TF expression. Sensitivity to hI-con1-dependent cell-mediated cytotoxicity (IDCC) was evaluated in 5-hour-chromium release assays. Finally, to investigate the effect of interleukin-2 (IL-2) on IDCC, 5-h ^51^Cr assays were also conducted in the presence of low doses of IL-2 (i.e., 50–100 IU ml^−1^).

**Results:**

Cytoplasmic and/or membrane TF expression was observed in all 16 (100%) USPC samples tested by IHC, but not in normal endometrium. High expression of TF was found in 50% (three out of six) of the USPC cell lines tested by real-time PCR and flow cytometry when compared with normal endometrial cells (NECs; *P*<0.001). Uterine serous papillary adenocarcinoma cell lines overexpressing TF, regardless of their high or low HER2/neu expression, were highly sensitive to IDCC (mean killing±s.d., 65.6±3.7%, range 57.5–77.0%, *P*<0.001), although negligible cytotoxicity against USPC was seen in the absence of hI-con1 or in the presence of Rituximab control antibody. The addition of low doses of IL-2 further increased the cytotoxic effect induced by hI-con1 against chemotherapy-resistant USPC.

**Conclusion:**

hI-con1 induces strong cytotoxicity against primary chemotherapy-resistant USPC cell lines overexpressing TF. The hI-con1 may represent a novel therapeutic agent for the treatment of patients harbouring advanced, recurrent and/or metastatic USPC refractory to standard treatment modalities.

Endometrial cancer is the most common female genital tract malignancy in the United States, with an incidence of 40 100 new cases and 7470 deaths in the United States in 2009 (Jemal *et al*, 2009). Two subtypes of endometrial carcinoma, namely type I and type II tumours, have been described on the basis of both clinical and histopathological variables (Bohkman, 1983). Type I endometrial cancers, which account for the majority of cases, are usually well differentiated and endometrioid in histology. These neoplasms are frequently diagnosed in younger women, are associated with a history of hyperestrogenism as the main risk factor, and typically have a favourable prognosis with appropriate therapy. In contrast, type II endometrial cancers include poorly differentiated endometrioid tumours (G3-endometrial endometrioid carcinoma), serous papillary (uterine serous papillary adenocarcinoma (USPC)) and clear cells endometrial carcinomas (Bohkman, 1983). Although these tumours account for a minority of endometrial cancers the striking majority of relapses and deaths occur in this group of patients (Hendrickson *et al*, 1982; Goff *et al*, 1994; Chan *et al*, 2003; Schwartz, 2006). The development of novel therapeutic strategies against this aggressive subset of endometrial cancers remains a high priority.

Uterine serous tumours, which accounts for about 10% of endometrial cancers, have a higher propensity for lymphovascular invasion, and intraperitoneal as well as extra-abdominal spread, than endometrioid carcinoma. At the time of presentation, approximately 60–70% of women with USPC will have disease spread outside the uterus (Hendrickson *et al*, 1982). Unlike the histologically similar high-grade ovarian cancer, USPC is a chemoresistant disease from onset, with *in vivo* responses to combined cisplatin-based chemotherapy in the order of 20% and of short duration (Hendrickson *et al*, 1982; Levenback *et al*, 1992; Chan *et al*, 2003). Our group has recently reported HER2/neu overexpression by immunohistochemistry (IHC) and also amplification of the *c-erbB2* gene by fluorescence *in situ* hybridisation in a large percentage of patients harbouring USPC (Santin *et al*, 2002, 2005a, 2005b, 2005c). These findings, recently confirmed by other groups (Diaz-Montes *et al*, 2006) including the Gynaecologic Oncology Group in a cooperative multicentric study (Grushko *et al*, 2008), have identified HER2/neu overexpression in USPC, as an independent variable associated with poor outcome and as the one that occurs more frequently in African–American women than in Caucasian women (Santin *et al*, 2002, 2005c).

Pathological angiogenesis, the formation of new capillary blood vessels from existing blood vessels into diseased tissues, has been previously reported to occur more frequently in endometrial carcinomas developing against a background of endometrial atrophy rather than carcinomas arising from a hyperplastic endometrium (Abulafia *et al*, 1999). Tissue factor, a transmembrane receptor for coagulation factor VII/VIIa, is aberrantly expressed in human cancers and on endothelial cells within the tumour vasculature (Contrino *et al*, 1996; Papetti and Herman, 2002). Importantly, tumour cells characterized by a high production of TF and vascular endothelial growth factor, a crucial initiator of angiogenesis, are known to generate solid tumours characterized by intense vascularity and highly aggressive behaviour (Abe *et al*, 1999). Consistent with this view, vascular endothelial growth factor expression at the invading tumour front is reported to be 4–10 times higher than in the inner tumour areas and is significantly associated with poor prognosis, particularly within stage I endometrial cancer (Abulafia *et al*, 1999; Bristow, 1999). Although a direct regulation of vascular endothelial growth factor expression in human tumour cells by the cytoplasmic tail of TF has been previously shown (Abe *et al*, 1999), recent studies indicate that type-2 proteinase activated receptor is intimately involved in TF-mediated signalling and angiogenesis (Ruf, 2007). These data suggest a potential direct role for TF in tumour growth (Ruf, 2007).

The hI-con1 is a previously characterized immunoconjugate molecule developed against TF (Hu *et al*, 1999; Hu and Garen, 2000, 2001). It is composed of two identical protein chains consisting of human Factor VII (fVII) as the targeting domain fused to human immunoglobulin (Ig) G1 Fc as the effector domain; the two chains are held together by the disulphide bonds normally present in IgG. The hI-con1 is designed to bind to TF with far higher affinity and specificity than that can be achieved with an anti-TF antibody. Indeed, the hI-con1 has several important advantages over monoclonal antibodies for targeting TF, including: (1) the Kd for fVII binding to TF is about 10^−12^ M (Waxman *et al*, 1992), in contrast to anti-TF antibodies that have a Kd in the range of 10^−8^–10^−9^ M for TF (Presta *et al*, 2001) and (2) the hI-con1 is produced by recombinant DNA technology, allowing a completely human hI-con1 to be made for future clinical trials. Because binding of the fVII to TF could induce disseminated intravascular coagulation, a potentially lethal vascular disease, an amino-acid substitution was introduced into the fVII domain of the hI-con1 (Lysine 341 to Alanine) to inhibit initiation of the coagulation pathway without reducing the strong affinity for TF (Hu *et al*, 1999; Dickinson *et al*, 2006). The human Fc domain of the hI-con1 may thus potentially activate powerful cytolytic responses mediated by antibody-dependent cell-mediated cytotoxicity against both TF-expressing tumour cells and tumour vascular endothelial cells that bind the hI-con1 molecule. In this study, we evaluated for the first time the *in vitro* potential of hI-con1 as a novel immunotherapeutic agent against biologically aggressive uterine serous tumours.

## Methods

### Tissue factor immunostaining of formalin-fixed USPC tissues

Formalin-fixed, paraffin-embedded tissue blocks from 16 patients harbouring stage I (6 patients), stage II (2 patients), stage III (6 patients) and stage IV (2 patients) USPC were retrieved from the surgical pathology files at Yale University. Specimens were reviewed by a surgical pathologist (NB). The level of TF expression was then evaluated on the most representative block by standard immunohistochemical staining. For IHC, 4 *μ*m sections were cut from the formalin-fixed paraffin-embedded blocks. After deparaffinization and rehydration, endogenous peroxidase was blocked in 3% H_2_O_2_. Steam and high pH (pH 9) were used for antigen retrieval. The slides were then incubated overnight at 4°C with monoclonal anti-TF antibody (no. 4509, 1 : 10 dilution; American Diagnostica, Stamford, CT, USA). EnVisionTM system (Dako, Carpinteria, CA, USA) was used for secondary detection and the reactions were visualised with diaminobenzidine. Appropriate positive and negative controls were used with each case. Both cytoplasmic and membranous immunoreactivity were considered positive. Immunostaining was assessed using a semi-quantitative scoring system, as follows: 0, negative (0–5% staining); 1+, weakly positive (10–20%); 2+, moderately positive (20–50%); 3+ and 4+, strongly positive (50–75% and >75%, respectively), as previously described (Kaushal *et al*, 2008).

### Establishment of USPC cell lines

Primary USPC tumour cell lines from six patients with invasive USPC were obtained from fresh tumour biopsies collected at the time of surgery, under the approval of the Institutional Review Board. Tumours were staged according to the International Federation of Gynaecologists and Obstetricians operative staging system. Six primary USPC cell lines (USPC-ARK-1, USPC-ARK-2, USPC-ARK-3, USPC-ARK-4, USPC-ARK-5 and USPC-ARK-6) were established after sterile processing of the tumour samples from surgical biopsies as described previously (Cross *et al*, 2010). Source–patient characteristics of these six USPC cell lines are described in Table 1. The amplification of the *c-erbB2* gene by fluorescence *in situ* hybridisation, expression levels of HER2/neu receptor by IHC and mRNA expression levels by quantitative real-time PCR (qRT–PCR) for these primary USPC cell lines have been recently reported (El-Sahwi *et al*, 2010).

### Quantitative real-time PCR

RNA isolation from all the six primary USPC cell lines used in the cytotoxicity experiments were performed using TRIzol Reagent (Invitrogen, Carlsbad, CA, USA) according to the manufacturer's instructions. Quantitative PCR was performed using a 7500 Real-time PCR System with the manufacturer's recommended protocol (Applied Biosystems, Foster City, CA, USA) to evaluate expression of TF in all samples. Each reaction was run in duplicate. Briefly, 5 *μ*g of total RNA from each sample was reverse transcribed using SuperScript III first-strand cDNA synthesis (Invitrogen). A total of 5 *μ*l of reverse transcribed RNA samples (from 500 *μ*l of total volume) was amplified using the TaqMan Universal PCR Master Mix (Applied Biosystems) to produce PCR products specific for TF. The primers and probe for TF were obtained from Applied Biosystems (assay ID Hs01076032_m1). The comparative threshold cycle (*C*_*T*_) method (Applied Biosystems) was used to determine gene expression in each sample relative to the value observed in the lowest non-malignant endometrial epithelial-cell sample, using glyceraldehyde-3-phosphate dehydrogenase (assay ID Hs99999905_m1) RNA as internal control.

### Flow cytometry

Clinical grade hI-con1 was produced for Iconic Therapeutics Inc. (Atlanta, GA, USA) by Laureate Pharma, (Princeton, NJ, USA) by cultivation of baby hamster kidney cells transfected with a vector containing the gene sequence originally described by Hu and Garen (2000, 2001; Hu *et al*, 1999). The protein was purified by a series of chromatographic steps to a purity adequate for clinical use, formulated in 15 mM HEPES, 150 mM NaCl, 5 mM CaCl_2_, 25 mM arginine and 0.01% Tween 80, pH 7.4 buffer, and was sterile filtered. It was provided in aliquots of 80 *μ*l at a stock concentration of 350 *μ*g ml^−1^, which were used for our flow cytometry and hI-con1-dependent cell-mediated cytotoxicity (IDCC) studies. Briefly, USPC cell lines and phytohemagglutinin (PHA)-stimulated control peripheral blood lymphocytes (PBLs) were stained with hI-con1 at a concentration of 30 *μ*g ml^−1^ for 30 min on ice or with a commercially available fluorescein isothiocyanate-conjugated mouse anti-human TF Ig (i.e., positive control; BioSource International, Camarillo, CA, USA). After hI-con1 staining, cells were washed twice with the same buffer and secondary goat-anti-mouse antibody (IgG1-FITC (fluorescein isothiocyanate), catalogue no. F0767 Sigma Aldrich, St Louis, MO, USA) was added for further 30 min. Analysis was conducted with a FACSCalibur instrument using Cell Quest software (Becton Dickinson, Franklin Lakes, NJ, USA).

### Tests for IDCC

A standard 5-h chromium (^51^Cr) release assay was performed to measure the cytotoxic reactivity of Ficoll–Hypaque-separated PBLs from several healthy donors in combination with hI-con1 against USPC target cell lines. The release of ^51^Cr from the target cells was measured, as described (Santin *et al*, 1999), as evidence of tumour cell lysis after exposure of tumour cells to various concentrations of hI-con1 (ranging from 1 to 80 *μ*g ml^−1^) and different target/effector cell ratios. Controls included the incubation of target cells alone or with PBLs, or monoclonal antibody (mAb) separately. The chimeric anti-CD20 mAb Rituximab (Rituxan; Genentech, San Francisco, CA, USA) was used as control in all bioassays. The hI-con1-dependent cell-mediated cytotoxicity was calculated as the percentage of killing of target cells observed with mAb plus effector cells as compared with ^51^Cr release from target cells incubated alone.

### Interleukin-2 enhancement of IDCC

To investigate the effect of IL-2 on IDCC, effector PBLs were incubated for 5 h at 37°C, with a final concentration of IL-2 (Aldesleukin; Chiron Therapeutics, Emeryville, CA, USA) ranging from 50 to 100 IU ml^−1^ in 96-well microtiter plates. Target cells were primary USPC cell lines exposed to hI-con1 (concentrations ranging from 1 to 80 *μ*g ml^−1^), whereas controls included the incubation of target cells alone or with PBLs in the presence or absence of IL-2 or mAb, respectively. Rituximab was used as a control mAb. The hI-con1-dependent cell-mediated cytotoxicity was calculated as the percentage of killing of target cells observed with mAb plus effector PBLs, as compared with target cells incubated alone. Each experiment was performed with PBLs collected from multiple normal donors and results from a representative donor are presented.

### Statistical analysis

For qRT–PCR data, the right-skewing was removed by taking copy-number ratios relative to the lowest expressing normal endometrial cell (NEC) sample (‘relative copy numbers’), log_2_ transforming them to ΔCTs, and comparing the results via unequal variance *t*-test for the USPC *vs* NEC difference. Group means with 95% confidence limits (confidence intervals) were calculated by computing them on the ΔCTs and then reverse transforming the results to obtain means (95% confidence intervals) of relative copy numbers. Differences in TF expression by flow cytometry were analysed by the unpaired *t*-test. Kruskal–Wallis test and *χ*^2^-analysis was used to evaluate differences in hI-con1-induced cellular cytotoxicity levels in primary tumour cell lines. Statistical analysis was performed using SPSS version 15 (SPSS, Chicago, IL, USA). A *P*-value of <0.05 was considered statistically significant.

## Results

### Tissue-factor expression by IHC in USPC samples

**Figure 1:**
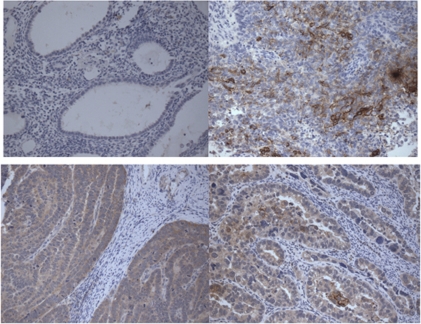
Representative immunohistochemistry localisation analyses of tissue factor (TF) in uterine serous papillary adenocarcinoma (USPC) specimens. Upper left panel: normal endometrium, which is negative for TF. Lower left panel: USPC specimen showing cytoplasmic expression of TF. Upper right panel: USPC specimen showing membrane expression of TF. Lower right panel: USPC specimen showing both cytoplasmic and membrane expressions of TF. Original magnification: × 200.

We performed immunohistochemical analysis of TF protein expression on formalin-fixed tumour tissues from 16 paraffin-embedded uterine serous carcinoma specimens. As representatively shown in Figure 1, with no exception, all USPC samples that were tested showed either membrane and/or cytoplasmic immunoreactivity for TF (i.e., all 16 samples=100%), whereas the non-neoplastic endometrial controls were negative. The staining intensity was focal (1+) in nine cases, moderately positive (2+) in four cases and strongly positive (3+) in two cases.

### Tissue-factor expression by qRT–PCR in USPC primary cell lines

A total of six primary USPC lines, half of which overexpress HER2/neu at 3+ level and show amplification of the *C-erbB2* gene by fluorescence *in situ* hybridisation, were tested for TF expression by qRT–PCR. Table 2 shows mRNA levels for TF in all USPC cell lines relative to the value observed in the lowest non-malignant endometrial epithelial-cell sample. Of the six tumours tested, three showed a high mRNA copy number (i.e., USPC-ARK-2, USPC-ARK-3 and USPC-ARK-6) ranging from 280 to 816 (Table 2). The TF expression between these USPC cell lines and NECs was statistically significant at *P*=0.01. The fold change in the mean relative copy numbers between high USPC TF expressers *vs* NECs was 8.7 (*P*=0.01). In contrast, low TF expression using qRT–PCR was detected in the remaining three USPC cell lines (USPC-ARK-1, USPC-ARK-4 and USPC-ARK-5; range of copies from 4 to 17; Table 2). The mean copy number in USPC samples overexpressing TF was 476.3 *vs* 12.3 in the low USPC TF expressers (*P*<0.01). High level of TF was detected in two out of three USPC cell lines harbouring the amplification of the *c-erbB2* gene and in one out of three USPC cell lines showing low HER2/neu expression (Table 2).

### Tissue-factor expression by flow cytometry in primary USPC cell lines

**Figure 2:**
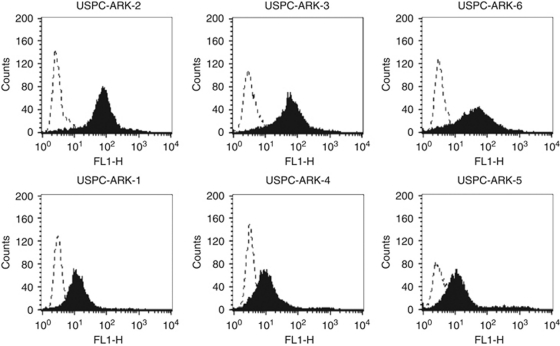
Flow cytometry histograms of primary uterine serous papillary adenocarcinoma (USPC) cell lines showing high (USPC-ARK-2, USPC-ARK-3 and USPC-ARK-6) and low (USPC-ARK-1, USPC-ARK-4 and USPC-ARK-5) expression of tissue factor. Dashed line represents isotype and solid black represents human immuno-conjugate molecule.

Surface TF receptor expression was evaluated by fluorescence-activated cell sorting analysis in all six primary USPC cell lines using hI-con1 and an anti-human TF control mAb. As negative controls, several PHA-stimulated PBLs established from healthy donors or the same USPC patients, from whom the tumour cell lines had been established, were also studied. In agreement with the RT–PCR results, high reactivity against TF was found using flow cytometry in USPC-ARK-2, USPC-ARK-3 and USPC-ARK-6 cell lines stained with hI-con1 (Table 2, Figure 2). In contrast, significantly lower TF surface expression was detected in USPC-ARK-1, USPC-ARK-4 and USPC-ARK-5 cell lines (Table 2, Figure 2). Mean fluorescence intensity ranged from 89 to 92 in high USPC TF expressers *vs* a mean fluorescence intensity ranged from 25 to 53 in low USPC TF expressers (*P*<0.02). Low TF expression was also observed in the multiple PHA-stimulated PBL lines used as further controls (i.e., mean fluorescence intensity from 40 to 45; high USPC TF expressors *vs* PHA-stimulated PBLs: *P*<0.01).

### Uterine serous papillary adenocarcinoma overexpressing TF are highly resistant to natural killer (NK) cell activity but sensitive to IDCC

**Figure 3:**
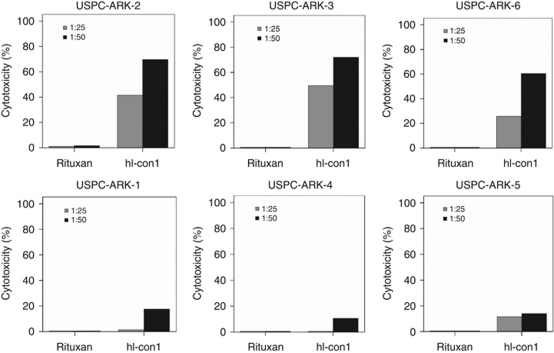
Representative cytotoxicity experiments using human immuno-conjugate molecule (hI-con1) against primary uterine serous papillary adenocarcinoma (USPC) with high *vs* low tissue factor (TF) expression. Upper panels: high TF USPC cell lines. Lower panels: low TF USPC cell lines. Negligible cytotoxicity was detected in the absence of hI-con1 or in the presence of rituximab control monoclonal antibody.

All the six primary USPC cell lines available were tested for their sensitivity to NK cell cytotoxicity when challenged with heterologous PBLs, collected from several healthy donors, in a standard 5-h ^51^Cr release assay. Uterine serous papillary adenocarcinoma primary cell lines were consistently found to be resistant to NK cell-mediated cytotoxicity when combined with PBLs at E : T ratios varying at 25 : 1–50 : 1 (range of cytotoxicity from 0.2–9% with all E : T ratios). Similarly, USPC cell lines incubated with rituximab (2.5 *μ*g ml^−1^) control antibody showed no significant cytotoxicity (range from 0.1–8%). We then investigated the sensitivity of USPC cell lines expressing different levels of TF to heterologous PBLs in the presence of hI-con1 (30 *μ*g ml^−1^). While negligible levels of cytotoxicity were detected against PHA-stimulated PBLs (i.e., TF-negative controls, data not shown), tumour cell lines expressing high levels of TF (i.e., USPC-ARK-2, USPC-ARK-3 and USPC-ARK-6), regardless of their high or low HER2/neu expression, were found to be highly sensitive to hI-con1-induced antibody-dependant cell death (mean±s.d., 65.6±3.7% range, 57.5–77.0% Figure 3; *P*<0.001). In contrast, tumour cell lines expressing low levels of TF (i.e., USPC-ARK-1, USPC-ARK-4 and USPC-ARK-5) were killed at significantly lower levels when compared with the high TF-expresser USPC cell lines (Figure 3; *P*<0.001). Nevertheless, in the latter experiments, low TF-expresser USPC cell lines incubated with hI-con1 were killed at significantly higher level when compared with the same tumours incubated with rituximab control mAb (mean hI-con1 killing low expresser USPC±s.d.=14.0±2.0% range, 10.6–17.6% Figure 3; *P*<0.02), or when compared with PHA-stimulated PBL control incubated with hI-con1 (mean killing PBLs=3.0% range, 0.6–6%).

### Interleukin-2 enhancement of IDCC against USPC

**Figure 4:**
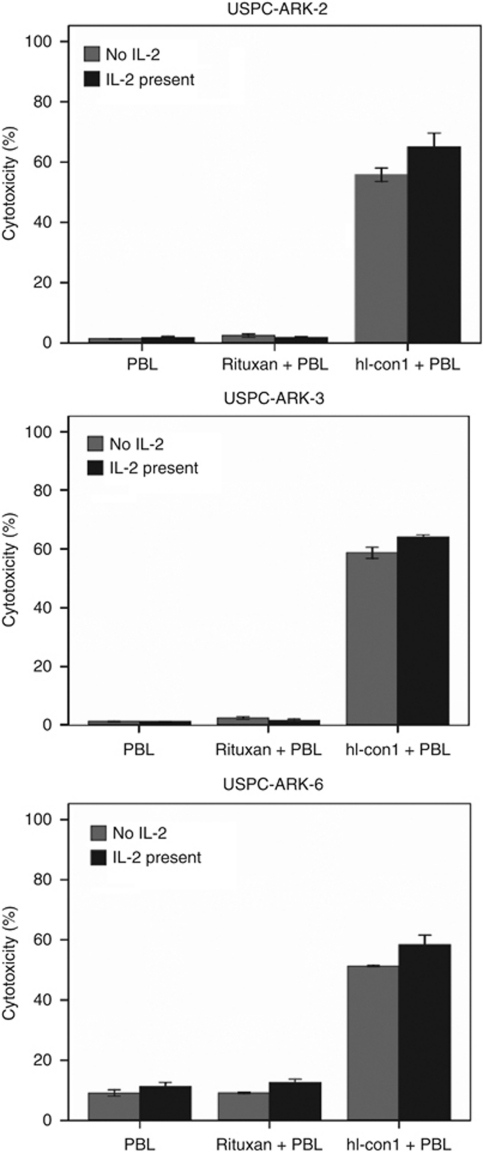
Effect of low doses of interleukin-2 (IL-2) in combination with human immuno-conjugate molecule (hI-con1; 30 *μ*g ml^−1^) on antibody-dependent cell-mediated cytotoxicity against USPC-ARK-2, USPC-ARK-3 and USPC-ARK-6 primary cell lines (effectors to target ratio 25 : 1). Peripheral blood lymphocytes (PBLs) from healthy donors were incubated for 4 h in the presence of 100 IU ml^−1^ of IL-2. The hI-con1-mediated antibody-dependent cell-mediated cytotoxicity was significantly increased in the presence of low doses of IL-2. No significant increase in cytotoxicity was detected after 4-h IL-2 treatment in the absence of hI-con1 or in the presence of the rituximab isotype control monoclonal antibody.

To investigate the effect of low doses of IL-2 in combination with hI-con1 (30 *μ*g ml^−1^) on IDCC against USPC cell lines, PBLs from healthy donors were incubated for 5 h in the presence of 50–100 IU ml^−1^ of IL-2. As shown in Figure 4, for all primary USPC showing TF overexpression, (i.e., USPC-ARK-2, USPC-ARK-3 and USPC-ARK-6) IDCC was increased in the presence of low doses of IL-2. Administration of 50–100 IU ml^−1^ of IL-2 to the effector PBLs at the start of the assay increased the cytotoxic activity against USPC cell lines overexpressing TF compared with the use of hI-con1 alone, (Figure 4; *P*< 0.01) whereas no significant increase in cytotoxicity was detected after 5-h IL-2 treatment in the absence of hI-con1 or in the presence of Rituximab control mAb (Figure 4). These results suggest that low levels of IL-2 may enhance hI-con1-mediated cell-cytotoxicity in USPC cell lines *in vitro*.

## Discussion

Tissue factor is involved in pathological angiogenesis and is abnormally overexpressed in multiple human tumours and in tumour vascular endothelial cells but not in normal quiescent vascular endothelial cells (Contrino *et al*, 1996; Papetti and Herman, 2002). Although TF, as a cell-surface receptor, is physiologically expressed on extravascular cells of many organs and in the adventitial layer of the blood vessel wall, it is sequestered by coagulation factor VII (fVII), a natural ligand for TF, at these sites by the tight endothelial-cell layer of the normal vasculature (Contrino *et al*, 1996; Papetti and Herman, 2002). Thus, pathologically expressed TF may provide a target for the development of novel cancer therapies that are effective not only against tumour cells but also against tumour blood vessels (Hu *et al*, 1999; Hu and Garen, 2000, 2001; Alessi *et al*, 2004).

In this study, we have evaluated TF expression using IHC in multiple formalin-fixed, paraffin-embedded USPC samples. In addition, we have studied TF expression at RNA and protein level in six primary USPC cell lines and subsequently tested the *in vitro* activity of hI-con1, a previously characterized immunoconjugate molecule developed against TF (Hu *et al*, 1999; Hu and Garen, 2000, 2001), as a novel therapy against these chemotherapy-resistant USPC cell lines *in vitro* (Cross *et al*, 2010). We observed TF overexpression on the membrane and/or in the cytoplasm of all the USPC samples tested using IHC (all 16=100%) whereas normal control endometrial cells were found consistently negative for TF expression. This suggests that TF expression may be a common and an important event in malignant transformation of the endometrium, particularly for biologically aggressive type II endometrial cancer. Consistent with this view, TF was also found to be highly expressed in three out of six primary USPC cell lines available for this study, using qRT–PCR and flow cytometry. The TF expression has been previously reported in endometrial endometrioid adenocarcinoma cell lines (i.e., the most common and less aggressive variant of human endometrial cancer; Kato *et al*, 2005, 2008). However, to our knowledge, the overexpression of TF in primary chemotherapy-resistant uterine serous tumours has not been previously studied. Of interest, in the primary USPC tested, both HER2/neu-positive and HER2/neu-negative cell lines were found to overexpress the TF. These data thus suggest that USPC, regardless of their different sensitivity/resistance to various chemotherapy agents (Cross *et al*, 2010) or anti-HER2/neu-targeted therapies, (i.e., trastuzumab and pertuzumab; El-Sahwi *et al*, 2010) may potentially respond to hI-con1 therapy.

Several studies have shown that tumour cell TF has an important role in tumour metastatic process, possibly by induction of the coating of the tumour cell with fibrin that would trap the cells in the microvasculature, thereby aiding metastases (Mueller *et al*, 1992). More recently, however, a possible direct role for TF in tumour growth has also been suggested by studies showing a dramatically reduced tumour growth in mice, where a selective reduction in TF was achieved using small interfering RNA (Yu *et al*, 2005). Of great interest, in these studies, is that the reduction in TF expression did not affect growth of the tumour cells *in vitro*, suggesting that TF-mediated enhancement of tumour growth requires a factor present *in vivo* that is not present when cells are grown *in vitro* (Yu *et al*, 2005). A potential candidate to explain these findings is therefore FVIIa, which would form a TF:FVIIa complex on the surface of tumour cells *in vivo* leading to the activation of type-2 proteinase activated receptor-dependent signalling (Ruf, 2007). These findings combined with our results suggest that TF overexpression may potentially provide an additional growth advantage to biologically aggressive USPC *in vivo*.

The potential cytotoxic activity of hI-con1 against human melanoma and prostate tumour cells has previously been shown by Hu *et al* (Hu *et al*, 1999; Hu and Garen, 2000, 2001). In our study, we extended the results of Hu *et al* (Hu *et al*, 1999) evaluating the cytotoxic potential of hI-con1 against multiple biologically aggressive, high-grade serous endometrial cancer cell lines. We found all tested primary tumours showing high TF expression, regardless of their high or low HER2/neu expression, to be highly susceptible to IDCC in the presence of effector cells. In this regard, it is worth noting that although these cell lines were resistant to NK-cell cytotoxic activity, IDCC resulted in killing of up to 65% of tumour cells in 5-h ^51^Cr release assays. Taken together, these *in vitro* results strongly suggest that TF may provide a novel target for USPC and its tumour vasculature, which should likely result in hI-con1-induced lysis of tumour cells as well as endothelial cells *in vivo*.

hI-con1-mediated tumour cell lysis depends on the presence of effector cells (Hu *et al*, 1999; Hu and Garen, 2000, 2001), and CD56-positive lymphocytes have been shown to contribute most to the effect of IgG1-mediated antibody-dependent cell-mediated cytotoxicity *in vitro* as well as *in vivo* (Ortaldo *et al*, 1987; Clynes *et al*, 2000). Interleukin-2 treatment leads to the activation of NK cell cytotoxicity and to the expansion of the NK cell population within the PBL *in vivo* (Fehniger *et al*, 2000). Consistent with its immunostimulatory effect on NK cells, IL-2 has been previously shown to work synergistically with monoclonal antibodies *in vivo* (Caron *et al*, 1995). Importantly, contrary to the significant toxicity of high-dose recombinant IL-2 therapy, low-dose IL-2 administered subcutaneously or by continuous infusion has been shown to have high clinical and immunological activity with a low-toxicity profile (Fehniger *et al*, 2000). These findings are particularly interesting because a modulation of the number and function of NK cells has been associated with tumour progression in both experimental and animal models, and pre-treatment of the PBLs with IL-2 can increase the cytotoxicity levels in patients with suppressed antibody-dependent cell-mediated cytotoxicity to levels similar to that in normal donors (Ortaldo *et al*, 1987). Consistent with this data, our *in vitro* experiments reveal a significant increase in hI-con1-induced cytotoxicity after the brief incubation of PBLs and tumour cells with IL-2 compared with the cytotoxicity induced by hI-con1 in the absence of IL-2. Interleukin-2 seems to, therefore, enhance the cytotoxic potential of the effector cells. The administration of low doses of IL-2 might therefore be a valid therapeutic option to increase IDCC in heavily pretreated endometrial cancer patients.

Although the *in vivo* use of hI-con1 in animals harbouring xenografts of human USPC has not been studied in our current report, previous studies have shown the safety and efficacy of using hI-con1 *in vivo* in mice carrying mouse and human tumours (Hu *et al*, 1999; Hu and Garen, 2000, 2001), harbouring human implants of pathological endometriosis (Krikun *et al*, 2010), as well as in eradicating choroidal neovascularization in mouse and pig models of wet-form macular degeneration (Tezel *et al*, 2007). Thus, current studies in multiple animal models suggest that hI-con1 use is potentially safe *in vivo* and, therefore, may have great potential as a neovascular targeting agent, with broad applications for the immunotherapy of pathological angiogenesis-involved diseases in humans.

In conclusion, expression of TF in chemotherapy-resistant USPC makes hI-con1 an attractive agent for immunotherapy of chemotherapy-resistant endometrial cancer. This holds particularly true for USPC patients, in whom other potentially effective targeted *in vivo* therapies (i.e., trastuzumab; Jewell *et al*, 2006; Villella *et al*, 2006; Santin *et al*, 2008) are not indicated owing to the lack of expression of its target HER2/neu on USPC cells. In this study, we have shown significant hI-con1-mediated killing of high-grade, chemotherapy-resistant USPC, for which treatment options are very limited. Human immuno-conjugate molecule (hI-con1) might therefore represent a fascinating new addition to the treatment of this aggressive disease and, potentially, multiple other human tumours overexpressing TF. Clinical trials will ultimately determine the validity of this novel therapeutic approach.

## Figures and Tables

**Table 1:** Patient characteristics, from which the six USPC cell lines were established

**Patient**	**Age (years)**	**Race**	**Year of diagnosis**	**FIGO stage**	**USPC histopathology**
USPC-ARK-1	62	AA	1997	IVA	Pure
USPC-ARK-2	63	AA	1998	IVB	Pure
USPC-ARK-3	59	AA	2006	IVB	Mixed
USPC-ARK-4	73	C	2004	IVB	Pure
USPC-ARK-5	73	AA	2006	IIIC	Pure
USPC-ARK-6	62	C	2005	IB	Mixed

Abbreviations: AA=African American; C=Caucasian; FIGO=International Federation of Gynaecologist and Obstetrics; USPC=uterine serous papillary adenocarcinoma.

**Table 2:** Tissue factor and HER2/neu expression in primary USPC cell lines

	**HER2/neu IHC**	**HER2/neu FISH**	**Tissue factor RT–PCR**	**Tissue factor flow cytometry**	
**Sample**	**Cell block**	**Cell block**	**mRNA copy number**	**% Gated**	**MFI**
Control	—	—	1	—	—
USPC-ARK-1	3+	2.5	14.6	86.7	32.7
USPC-ARK-2	3+	5.2	332.3	100.0	92.2
USPC-ARK-3	3+	4.7	280.2	100.0	89.8
USPC-ARK-4	0	1.6	4.7	75.6	25.8
USPC-ARK-5	0	1.4	17.6	55.3	53.6
USPC-ARK-6	1+	0.9	816.4	100.0	92.5

Abbreviations: FISH=fluorescence *in-situ* hybridization; IHC=immunohistochemistry; MFI=mean fluorescence intensity; RT–PCR=real-time PCR; USPC=uterine serous papillary adenocarcinoma.
